# Successful Extracorporeal Membrane Oxygenation (ECMO) Use without Systemic Anticoagulation for Acute Respiratory Distress Syndrome in a Patient with Aneurysmal Subarachnoid Hemorrhage

**DOI:** 10.1155/2019/9537453

**Published:** 2019-07-10

**Authors:** Amanda L. Faulkner, James David Bacon, Brian A. Fischer, Stephen L. Grupke, Kevin W. Hatton

**Affiliations:** ^1^Department of Anesthesiology, N-202 UKMC, 800 Rose Street, Lexington, KY 40536, USA; ^2^Department of Neurosurgery, 800 Rose Street, Lexington, KY 40536, USA

## Abstract

Extracorporeal membrane oxygenation (ECMO) is an important life-saving technology for patients with severe acute respiratory distress syndrome (ARDS). Unfortunately, ECMO has been traditionally contraindicated in patients with hemorrhagic neurologic diseases. The recent improvement in ECMO devices, increased utilization and experience with venovenous ECMO technologies among healthcare teams, and the use of ECMO without anticoagulation has expanded the potential populations that may benefit from ECMO. We present a case of successful utilization of venovenous ECMO for severe respiratory failure secondary to ARDS in a patient with aneurysmal subarachnoid hemorrhage and severe, episodic cerebral vasospasm. We also discuss important limitations and considerations for future successful use of ECMO in hemorrhagic stroke. This case report highlights the potential for this life-saving technology in patients with hemorrhagic stroke.

## 1. Introduction

Acute respiratory distress syndrome (ARDS) is a life-threatening respiratory system clinical presentation resulting in severe hypoxemia that frequently requires mechanical ventilation and adjunctive respiratory therapies to support respiratory function [1]. Venovenous extracorporeal membrane oxygenation (VV-ECMO) is an evolving therapy that is sometimes used as rescue in patients with severe ARDS to stabilize gas exchange until lung recovery occurs [2–6]. VV-ECMO was developed from cardiopulmonary bypass (CPB) technologies and its use has historically required complicated CPB devices, surgical and perfusion technical expertise, and systemic anticoagulation [6–8]. For these reasons, the use of VV-ECMO has been contraindicated in hemorrhagic neurologic diseases, such as aneurysmal subarachnoid hemorrhage (aSAH) [8–10]. The development of less-complicated VV-ECMO devices, the increased experience with VV-ECMO technologies among critical care specialists, critical care nurses, and other support staff, and the growing expertise in the use of VV-ECMO without anticoagulation have expanded the potential populations that may benefit from VV-ECMO. We present a case of successful utilization of VV-ECMO without systemic anticoagulation for severe respiratory failure in a patient with aSAH. The patient has provided written consent to publish this case report.

## 2. Case Report

A 44-year-old female with history of asthma, tobacco abuse, intravenous drug abuse, schizophrenia, and chronic hepatitis C infection was transferred to our hospital after computed tomography (CT) scan at an outside hospital demonstrated aSAH. During transport, she had an acute decline in her mental status and was emergently intubated after several failed attempts on arrival to our emergency department. Additional imaging revealed an anterior communicating artery aneurysm with surrounding subarachnoid hemorrhage, intraventricular extension, and hydrocephalus. An extraventricular drain (EVD) was placed emergently and she underwent uncomplicated endovascular coiling of her aneurysm that same day.

Following her coiling procedure, she developed worsening oxygenation and vasodilatory shock, presumed to be from aspiration pneumonitis as she had negative respiratory cultures, a negative procalcitonin analysis, and mildly hyperdynamic left ventricular function on cardiac echocardiography. On post-bleed day (PBD) 1, she remained on mechanical ventilation, her chest x-ray demonstrated bilateral infiltrates, her PaO2/FiO2 ratio was less than 100, and her clinical course was consistent with severe Acute Respiratory Distress Syndrome (ARDS) [11]. Lung-protective ventilation was initiated with increased oxygen (FiO2), positive end-expiratory pressure (PEEP), and inhaled epoprostenol therapy as per protocol [12]. On PBD 2, despite neuromuscular blockade, inhaled epoprostanol, and maximal ventilator support (FiO2=100%, PEEP=15 cmH20) which was limited by her hemodynamic response to additional PEEP, the patient developed refractory hypoxemic respiratory failure and VV-ECMO was proposed to support her worsening lung function. After a multidisciplinary discussion that included the patient's family, neurosurgery (NS), critical care medicine (CCM), and mechanical circulatory support (MCS) teams, the decision was made to proceed with bedside cannulation for VV-ECMO to facilitate respiratory support.

In the Neuroscience Intensive Care Unit (NSICU), the MCS team placed a 25 French ECMO access cannula in the right femoral vein (outflow to ECMO) and an 18 French ECMO return cannula in the left subclavian vein (inflow from ECMO) without difficulty. The internal jugular vein was not utilized for this cannulation due to concern for obstruction to intracranial venous outflow. VV-ECMO was then initiated using a CARDIOHELP ECMO System (Maquet Cardiovascular, Wayne, NJ). Due to concern for additional subarachnoid hemorrhage, routine heparin bolus was not administered at the time of ECMO initiation and therapeutic anticoagulation was not administered at any time during her VV-ECMO therapy. After VV-ECMO initiation, her hypoxemia and hypercarbia rapidly stabilized and ventilator settings were reduced to provide additional lung protection. This was accomplished by converting mechanical ventilation from a volume-control mode to a pressure-control mode with target tidal volume maintained between 4 mL/kg and 6 mL/kg and measured plateau pressure less than 30 cm H2O (Table 1). She was then transported to the Cardiovascular Intensive Care Unit (CVICU) for treatment and monitoring during VV-ECMO therapy. Daily multidisciplinary rounds with NS, CCM, and MCS teams evaluated her neurologic and other organ system injuries and codeveloped a daily plan of care. Daily evaluation of plasma free hemoglobin and routine evaluation for the development of thrombosis in the oxygenator and tubing were performed by perfusion, nursing, and physician MCS specialists. VV-ECMO blood flow rate was maintained greater than 3 LPM to reduce the risk of thrombosis and VV-ECMO FiO2 and VV-ECMO sweep gas settings were titrated to maintain systemic pH > 7.25, PaO2 55-80 mmHg, and PaCO2 35-50 mmHg.

Unfortunately, on PBD 5, she developed acute kidney injury that required initiation of continuous renal replacement therapy (CRRT) via her femoral VV-ECMO cannula. On PBD 6, her transcranial Doppler (TCD) values significantly increased and, following a multidisciplinary conversation, she was transported to the angiography suite where CV was identified and treated with intra-arterial verapamil. During angiography, she developed significant hypoxemia requiring increased ventilator and ECMO support to maintain adequate oxygenation (Table 1). Her hypoxemia improved on return to the CVICU and her ECMO and ventilator support were returned to minimal settings after approximately 24 hours. Because of the risk for additional CV and the potential for recurrent hypoxemia during angiography and vasodilator administration, the multidisciplinary team decided to continue ECMO therapy through at least PBD 10. On PBD 9, her TCD values again increased and she was transported back to the angiography suite for additional diagnostic angiography and intra-arterial vasodilator administration. No hypoxemia occurred during this second procedure.

On PBD 10, after a seven-day course of VV-ECMO, her respiratory status had significantly improved, she had not developed additional hypoxemia after angiography, and she required only minimal VV-ECMO and ventilator settings. After another multidisciplinary conversation, she was successfully separated from VV-ECMO and her venous cannulae were removed. Importantly, there was no evidence of enlargement of her subarachnoid hemorrhage. On PBD 11, she underwent percutaneous tracheostomy, percutaneous enterogastrostomy (PEG) tube, and tunneled dialysis catheter placement. On PBD 22, she underwent placement of a permanent ventriculoperitoneal shunt. On PBD 36, she was transferred from the NSICU to a long-term acute care hospital for continued ventilator weaning and aggressive neurorehabilitation. At the time of her transfer, she opened her eyes spontaneously, followed verbal commands with all four extremities and attempted phonation during tracheostomy collar trials. At her 90-day clinic followup, her tracheostomy had been removed and she was alert, oriented, and able to converse with appropriate syntax and content and was independently ambulatory without assistance. Her venous cannulation sites, arterial angiography access sites, and ventriculoperitoneal shunt surgery wounds were well-healed.

## 3. Discussion

In this case report, we highlight the successful use of VV-ECMO to support respiratory failure with severe ARDS, likely resulting from aspiration pneumonitis, in a patient with aneurysmal subarachnoid hemorrhage. In this case, we learned the importance of a multidisciplinary approach to this complicated technology in a high-risk patient, the necessity to plan for transport throughout the hospital during ECMO therapy, especially to areas not traditionally used by patients with ECMO, such as the angiography suite, and the potential to use VV-ECMO for a prolonged period of time without systemic anticoagulation.

Despite significant preclinical and clinical research to develop new therapies for ARDS, the management of patients with ARDS remains largely supportive. [1]. Current ARDS support therapies include lung protective ventilation strategies, prone positioning, meticulous attention to fluid balance, and early recognition and reduction of secondary injuries, including malnutrition, pneumonia, and deep venous thrombosis [13, 14]. The use of VV-ECMO to support the failing respiratory system in ARDS remains controversial [15]. While early studies of ECMO, primarily as a rescue therapy in severe ARDS, failed to demonstrate significant benefit to patients, the development of technical improvements and strategies that leaned toward earlier initiation of ECMO support improved clinical outcomes of ECMO in ARDS [16]. In 2009, the CESAR trial demonstrated, for the first time, that the use of ECMO as an early rescue therapy provided the potential for benefit in patients with severe ARDS [17]. Subsequent studies, including the recently-published EOLIA trial, have continued to refine our knowledge and clinical experience V-V ECMO in populations with ARDS [18]. While research is ongoing, it is increasingly apparent that V-V ECMO can be a viable option for many patients with ARDS, including populations that may have, traditionally, been excluded from this life-saving therapy.

aSAH is a devastating acute hemorrhagic neurologic disease affecting approximately 30,000 Americans every year. Between 30 and 50% of these patients will have significant long-term neurologic disability. aSAH occurs when an intracranial arterial aneurysm ruptures, releasing oxygenated blood into the subarachnoid space. Immediate complications from aneurysm rupture include severe neurologic injury with frequent loss of consciousness, hydrocephalus, seizure and neurocardiogenic shock. The primary goal of immediate therapy includes aneurysm obliteration, stabilization of cardiopulmonary status, and extracranial drainage of cerebrospinal fluid (CSF). Cerebral vasospasm (CV) is a secondary neurologic insult that occurs 3-10 days after aneurysm rupture and results in delayed cerebral ischemia, cerebral infarction and additional neurologic disability. Patients with aSAH are monitored closely for CV for approximately 2 weeks after their aneurysm rupture and undergo digital subtraction angiography with injection of intra-arterial vasodilators to spastic arteries when CV is detected.

In patients with aSAH, as is true of many critically ill patients with neurologic injury, ventilator management consists of balancing support of the failing lungs and the injured brain. Treatments for hypoxemia, including increasing FiO2, PEEP, and diuresis, may all be associated with negative consequences in patients with aSAH. Treatments for hypercarbia, including increasing respiratory rate and tidal volume, may also be associated with negative consequences in patients with aSAH. Likewise, permissive hypercapnia may not be possible in patients with elevated intracranial pressure. Given these potential problems, the decision to pursue alternatives to mechanical ventilation, such as VV-ECMO therapy, may be desirable to balance the management of respiratory and neurologic injury in these critically ill patients.

In our institution, our neurocritical care team is staffed by anesthesiology critical care specialists who also rotate their clinical service in the cardiovascular intensive care unit (CVICU) and surgical intensive care unit (SICU). Because of this “cross-training” across the NSICU, the CVICU, and the SICU, the critical care team has developed significant expertise in team-based management of VV-ECMO for patients with various cardiac, pulmonary, and cardiopulmonary diseases. This expertise was utilized to facilitate shared decision-making among the care teams and to bridge physician, nursing, respiratory therapy, and other healthcare worker knowledge gaps in the care of patients with aSAH and the utilization of VV-ECMO.

In addition to the multidisciplinary approach, our team also learned to plan for patient transport throughout the hospital, including to the angiography suite. In our institution, the angiography suite is in a different, but connected, hospital building. To transport the patient from the ICU to angiography, the patient traveled via an elevator and several hallways. This process took up to 30 minutes, depending on the time of day, and utilized physician, nursing, perfusion, and respiratory therapist expertise to safely transport this patient back and forth from the angiography suite. Prior to transport, contingency plans were developed and additional staff and resources were prepositioned along the route to assist with unexpected problems that may have occurred during transport. Because of these actions, the patient did not have any significant issues or problems during any of transports to or from angiography.

Finally, in this case we were able to utilize VV-ECMO for approximately 1 week without systemic anticoagulation. The major concerns with this strategy were increased risk of clot formation in the oxygenator and venous thromboembolism in the patient [10]. To reduce these risks, short lengths of heparin-bonded VV-ECMO tubing was used for this patient [1]. In addition, the VV-ECMO pump speed was maintained at a slightly higher than normal speed for increased flow throughout the VV-ECMO circuit. Fortunately, routine evaluation of the oxygenator did not demonstrate clot formation during VV-ECMO therapy, there were no significant changes in daily plasma free hemoglobin levels, and the patient did not exhibit signs of venous thromboembolism.

While it is not the first reported use of VV-ECMO in a patient with aSAH, our case was substantially different from the previously reported case and significantly adds to the overall experience of the use of VV-ECMO in patients with aSAH [19]. In the original case, the patient deteriorated within a few hours of aneurysm rupture from apparent neurogenic pulmonary edema, underwent VV-ECMO cannulation in the operating room immediately prior to a surgical clipping procedure, suffered severe intracranial hypertension, required pharmacologic coma, and was not monitored or treated for CV during hospitalization. Unlike our case, the patient was placed on nafamostat mesylate, a serine protease inhibitor most frequently used as an anticoagulant during hemodialysis.

In summary, we report the successful use of VV-ECMO to support severe respiratory failure secondary to ARDS in a patient with aSAH who underwent endovascular coiling and developed multiple episodes of CV during her hospitalization. Our report highlights the importance of a multidisciplinary approach among physician, nursing, pharmacy, perfusion, respiratory therapy, and many other experts, as well as, the importance of understanding transportation issues for these complex medical patients. In addition, it further highlights the growing experience with the use of VV-ECMO without systemic anticoagulation in patients with hemorrhagic neurologic disease. Future cases should consider these lessons and apply ECMO in appropriate patients with multidisciplinary support to achieve maximum patient benefit.

## Figures and Tables

**Table 1:** Extracorporeal Life Support (ECMO) and Mechanical Ventilation Parameters. ECMO was initiated on PBD 2 and discontinued on PBD 10. PBD = Post-bleed day. PEEP = Positive end-expiratory pressure.

PBD	ECMO FiO2 (%)	ECMO Sweep (L/min)	ECMO Flow (L/min)	Ventilator FiO2 (%)	Ventilator Mode	Pressure Above PEEP/PEEP (cm H2O)	pH	PaO2	PaCO2
2	100	3	3.9	100	PCV	15/10	7.30	244	54
3	100	6	4.1	50	PCV	15/10	7.45	152	36
4	70	6	3.9	50	PCV	15/10	7.47	124	34
5	30	1	3.7	50	PCV	12/8	7.43	97	36
6	21	0	3.8	50	PCV	20/5	7.34	84	38
7	60	3	6.5	60	PCV	18/10	7.42	76	46
8	21	0	3.5	40	PSV	10/10	7.36	117	46
9	21	0	3.6	40	PSV	12/8	7.38	95	42
10	21	0	3.5	40	PSV	10/5	7.43	84	36
